# Candidate genes associated with fatty acid compositions in north American Atlantic salmon (*Salmo salar*)

**DOI:** 10.1186/s12864-024-11131-2

**Published:** 2024-12-18

**Authors:** Barbara L. Langille, Manuel Juárez, Nuria Prieto, Solomon Boison, Panya Sae Lim, Bruce D. Swift, Amber F. Garber

**Affiliations:** 1 The Huntsman Marine Science Centre, 1 Lower Campus Rd., St. Andrews, NB E5B 2L7 Canada; 2 Agriculture and Agri-Food Canada Lacombe Research and Development Centre, 6000 C and E Trail, Lacombe, AB T4L 1W1 Canada; 3 Mowi Genetics AS, Sandviksbodene 77A, Bergen, 5035 Norway; 4 Tri-Gen Fish Improvement Ltd., Site 13 Comp 27 RR1, Lacombe, AB T4L 2N1 Canada

**Keywords:** Fatty acid, Omega, Selective breeding, *fads2*

## Abstract

Fatty acids are a requirement for normal development, however, since humans are unable to de novo produce essential fatty acids, they must be obtained from diet. Atlantic salmon is a major dietary source of nutritious and digestible fatty acids. Here, we set out to uncover the genomic basis of individual fatty acids and indices (saturated fatty acids, monounsaturated fatty acids, polyunsaturated fatty acids, n-3, and n-6) in 208 North American Atlantic salmon, to understand selection potential toward increasing relative quantities of essential fatty acids and to identify candidate genes for future research. Total n-6 (pro-inflammatory) was higher than total n-3 (anti-inflammatory) fatty acids with a ratio of 1 : 1.31 (n-3 : n-6). Heritability of fatty acids ranged from 0 to 0.99, however, most fatty acids and indices had moderate to high heritabilities (ranged from 0.20 to 0.88), implying that selection for improvement of traits could be possible. We found the same significant markers on chromosome 23 (based on false discovery rate thresholds of 2.0e-6 and suggestive significant thresholds of 2.0e-5 in Manhattan plots) in four fatty acids (γ-linoleic acid, stearidonic acid, dihimo-γ-linolenic acid, and eicosatrienoic acid), where three genes (*sin3b*, *acbd6*, and *fads2*) are known to be involved in lipid metabolism. These genes, *fads2* in particular, would all make ideal candidates for future functional studies. In addition, there were four fatty acids with loci over the suggestive significant threshold with a variety of markers on different chromosomes (lauric acid, stearic acid, eicosatetraenoic acid (ETA), and docosadienoic acid), with associated genes that had relevant functions to fatty acids or adipose cells in general.

**Supplementary Information:**

The online version contains supplementary material available at 10.1186/s12864-024-11131-2.

## Introduction

Atlantic salmon (*Salmo salar*) is an essential global protein source [1]. Industries of important aquaculture species, such as Atlantic salmon, have been establishing increasingly sustainable practices while improving fish quality by the use of selective breeding (either based on pedigree or genotypes). Most selective breeding effort has been put into growth gains and stress tolerance (such as temperature or sea lice) as these traits quickly translate into increased available protein and improved welfare [2, 3]. However, salmon, and marine fish in general, are also a major dietary source of nutritious and digestible fatty acids (FAs) [4, 5] despite a noted decrease in essential FAs in salmon fish fillets over the past few decades, often linked with the change of fish feed from fishmeal to a plant-based diet [6, 7]. Therefore, some breeding programs have also added improvement of FA composition, specifically omega-3 (n-3), to their selection strategy [8], with early results suggesting specific genetic factors may be responsible for specific FAs in salmon [4, 5, 9] and a variety of fish species [10, 11].

There are three categories of naturally occurring FAs: saturated FAs (SFAs), monounsaturated FAs (MUFAs), and polyunsaturated FAs (PUFAs), where further classification of PUFAs can be made depending on the position of the double bond on methyl terminal (which denotes the omega variety). The two most common PUFAs, n-3 and n-6 FAs, are unable to be produced within the human body, and therefore must come from the diet [12]. Long chain PUFAs (contain > 12 carbon atoms) are converted to very long chain PUFAs (contain 22 + carbon atoms) with the help of fatty acyl-CoA synthetases, Δ−5 and Δ−6 desaturases, and respective elongases (ELOVL). Decades of research has shown the many health benefits of n-3 and n-6 FAs, with a balanced intake of both being a requirement for homeostasis and normal development [13–15], as these two omega groups have opposite effects in the body (n-3 having anti-inflammatory roles whereas n-6 is inflammatory) [16, 17]. Anti-inflammatory properties of n-3 are important for wound healing and reducing the risk of certain diseases and cancers, while pro-inflammatory properties of n-6 are important for protecting the body against certain pathogens, infections, and injury.

The majority of studies trying to uncover the underlying genetic architecture of FAs have been performed in cattle [18–20] and pigs [21–23] with results indicating that genomic selection should be possible based on moderate to high heritabilities of FAs, and promising candidate genes. However, in recent years, studies have emerged investigating genomic associations with n-3 FAs and fillet fat content in Atlantic salmon [4, 24]. Horn et al. [4] investigated FAs of the n-3 pathway where they reported no genome wide significant loci but markers that exceeded the suggestive significant thresholds in two genomic regions on chromosome 21. When they further investigated the ratio of docosahexaenoic acid (DHA) to docosapentaenoic acid (DPA), they found significant markers that were located on chromosome 19, not far from the *elovl2* gene. The *elovl2* gene is important in the conversion of DPA to DHA, therefore, this gene makes an excellent candidate for future research. Recently, Harvey et al. [24] found all three PUFA desaturases (fads2d5, fads2d6a and fads2d6b), several lipid and FA transporters, and FA synthase associated with fillet fat content. These two studies were conducted using European origin Atlantic salmon and it is therefore, of interest to study the North Atlantic origin Atlantic salmon population, given the different number of chromosomes between these sub-populations.

The terrestrial meat industry, specifically beef, pork and poultry, has focused on identifying FA content in their products, but this has not been the case for many aquaculture species. It is expected that as aquaculture continues to expand, the value of products based on these metrics will likely become important in the future. Therefore, the aim of the current study was to uncover QTLs for FA composition and content in North American Atlantic salmon fillets and to identify possible gene targets for future studies.

## Methods

### Breeding program and harvest evaluation

The Huntsman Marine Science Centre (Huntsman; St. Andrews, New Brunswick, Canada) utilized 56 dams and 38 sires of Saint John River Atlantic salmon to produce 90 families in a partial factorial mating design, which became the 2011 year class (YC) of a commercial breeding program. All families were combined post hatch and communally reared in a commercial hatchery facility. Approximately 11,500 fish were stocked into a single sea cage in Granger Cove, New Brunswick, Canada for commercial grow out by Northern Harvest Sea Farms Ltd (see [25] for more details). Appropriately sized diet (Skretting, Canada) was fed each life stage of the salmon using their recommended diet tables for commercial hatchery and sea cage production.

The harvest evaluation occurred over two separate days, where harvested fish were placed in large tubs. Six or seven fish were randomly taken from each tub for an assessment of the fish throughout the entire sea cage. Fin clips were collected, and a unique number was assigned to each fish to genetically assign individuals back to families, as fish were not previously tagged due to small size when combined. Carcass traits, including bled weight (g), fork length (cm), head length from tip of snout to medial edge of operculum (cm), and gonad weight (g), were recorded one day post-harvest. Maturity and sex were verified in each fish. Fillet traits, including fillet weight (g), melanin discoloration (melanin and black spots), gaping (tears between muscle layers), marbling (variation across the fillet), and fillet color (assessed using a Roche SalmoFan and a Minolta Chroma Meter CR-410 C illuminate), were recorded on the second day post-harvest following *rigor mortis*. With the exception of fillet weight being completed on both fillets, all other fillet traits were recorded using only the right fillet.

### Fatty acid analysis

A total of 208 salmon from the harvest evaluation were selected based on perceived variation (i.e. wide range of color from the harvested fish) and a portion of the fillet was labelled, vacuum packaged, frozen and then shipped to the Agriculture and Agri-Food Canada-Lacombe Research and Development Centre (Lacombe, AB). Upon arrival in Lacombe, flesh samples from the left fillet were stored at −35 °C until they could be analyzed. Samples were analyzed over a one-week period, completing approximately 40 per day. On the day prior to analysis, samples were removed from the freezer, frozen weight recorded (the weight of the bag was corrected for) and placed in a refrigerator to thaw for 24 h. Samples were then removed from vacuum package, weighed, ground, and assigned a unique Lacombe ID to be used as identifier through the remainder of sample analysis. Lipids were then extracted using 2:1 chloroform: methanol, using a solvent to sample ratio of 20:1 [26]. Chloroform extracts were dried and dissolved in toluene, and lipids were methylated with 5% methanolic HCl [27]. A total of 35 FA methyl esters (FAME) were extracted into hexane, dried over anhydrous sodium sulfate and analyzed using the gas chromatography equipment and conditions described by Dugan et al. [28]. The FAs were expressed as a percentage of total FA and milligram (mg) FA per 1 gram (g) of tissue. Total SFA, MUFA, PUFA, n-3 (anti-inflammatory) and n-6 (pro-inflammatory) were then calculated.

### Pedigree variance component estimations

The following linear animal mixed model in ASReml-R 4.1 (*asreml* flag in *asreml* package) [29] in R version 4.3.0 [30] was used to estimate the genetic parameters and heritability for absolute content of fatty acids:

$$\:{y}_{ijklm}=\:\mu\:+{\text{s}\text{e}\text{x}}_{i}+{\text{m}\text{a}\text{t}\text{u}\text{r}\text{i}\text{t}\text{y}}_{j}+{\beta\:}_{1}{\text{w}\text{e}\text{i}\text{g}\text{h}\text{t}}_{k}+{\beta\:}_{2}{\text{t}\text{o}\text{t}\text{a}\text{l}\text{f}\text{a}\text{t}}_{l}+\:{a}_{m}+{e}_{ijklm}\:,$$

where $\:y$ is the data of the phenotypic measurements, $\:\mu\:$ is the overall mean, $\:{\text{s}\text{e}\text{x}}_{i}$ is the fixed effect of observations (*i* = 1: male, 2: female, 3:unknown), and $\:{\text{m}\text{a}\text{t}\text{u}\text{r}\text{i}\text{t}\text{y}}_{j}$ is the fixed effect corrected for maturity (*j* = 1: immature fish, 2: fish that is undergoing maturation and will spawn later in the same year as assessment - determined by assessing the gonads), $\:{\text{w}\text{e}\text{i}\text{g}\text{h}\text{t}}_{k}$ and $\:{\text{t}\text{o}\text{t}\text{a}\text{l}\text{f}\text{a}\text{t}}_{l}$ (i.e. the total amount of fat present in each fillet) are the fixed covariates included in the model to account for the relationship between phenotype traits and body weight or total fillet fat (both important covariates that have been shown to impact FA results) of the *k*^th^ or *l*^th^ individual, where $\:{\beta\:}_{1}$and $\:{\beta\:}_{2}$ are the regression coefficients. The $\:{a}_{m}$ is the random additive genetic effect, $\:\varvec{a}$ ~ *N*(0, **A**σ_*a*_^2^) of the *m*^th^ animal, where **A** is the numerator relationship matrix, and $\:{e}_{ijklm}$ is the random residual effect, ***e*** ~ *N*(0, **I**σ_*e*_^2^), where **I** is the identity matrix. The same model was used for proportional content, however, $\:{\beta\:}_{2}{\text{t}\text{o}\text{t}\text{a}\text{l}\text{f}\text{a}\text{t}}_{l}$ was removed. Fixed effects were evaluated for significance using a Wald test with the *wald.asreml* flag. Narrow sense heritabilities (*h*^2^) were estimated using *vpredict* in the ASReml package as *h*^2^ = σ_*a*_^2^ / (σ_*a*_^2^ + σ_*e*_^2^), where σ_*a*_^2^ and σ_*e*_^2^ were the genetic and residual variances, respectively.

We computed and estimated the phenotypic and genetic correlations between all traits (35 FAs, weight, and total fillet fat). The function *pairs.panels* in the *psych* package in R [31] was utilized to compute the phenotypic correlations. *Pairs.panels* creates a data matrix with Pearson correlations above the diagonal, bivariate scatterplots below the diagonal, and histograms on the diagonal. A bivariate linear mixed animal model in ASReml-R was used to estimate the genetic correlations between all traits:

$$\:\left[\begin{array}{c}{\varvec{y}}_{1}\\\:{\varvec{y}}_{2}\end{array}\right]=\left[\begin{array}{cc}{\mathbf{X}}_{1}&\:0\\\:0&\:{\mathbf{X}}_{2}\end{array}\right]\left[\begin{array}{c}{\mathbf{b}}_{1}\\\:{\mathbf{b}}_{2}\end{array}\right]+\left[\begin{array}{cc}{\mathbf{Z}}_{1}&\:0\\\:0&\:{\mathbf{Z}}_{2}\end{array}\right]\left[\begin{array}{c}{\mathbf{a}}_{1}\\\:{\mathbf{a}}_{2}\end{array}\right]+\left[\begin{array}{c}{\mathbf{e}}_{1}\\\:{\mathbf{e}}_{2}\end{array}\right],$$

where, $\:{\varvec{y}}_{1}$ and $\:{\varvec{y}}_{2}$ are the data vectors of the phenotypic measurements of interest; $\:{\mathbf{X}}_{1}$ and $\:{\mathbf{X}}_{2}\:$are the incidence matrices of the fixed effects and covariates as described in the univariate animal mixed model above (weight and total fat were added as covariates when they were not the response variable (traits) in the above model); $\:{\mathbf{b}}_{1}$ and $\:{\mathbf{b}}_{2}$ are the solution vectors for corresponding fixed effects; $\:{\mathbf{Z}}_{1}$ and $\:{\mathbf{Z}}_{2}\:$are the incidence matrices of the random animal effects; $\:{\mathbf{a}}_{1}$ and $\:{\mathbf{a}}_{2}$ are additive genetic effects for the first and second phenotypes following multivariate normal distribution (*MVN*) for the pedigree relationship matrix (**A**):

$$\:\begin{bmatrix}{\mathbf a}_1\\\:{\mathbf a}_2\end{bmatrix}\sim MVN\left(\begin{bmatrix}0\\\:0\end{bmatrix},\begin{bmatrix}{\sigma\:}_{{\text{a}}_1}^2&\:{\sigma\:}_{{\text{a}}_1,{\text{a}}_2}\\\:{\sigma\:}_{{\text{a}}_1,{\text{a}}_2}&\:{\sigma\:}_{{\text{a}}_2}^2\end{bmatrix}\otimes\:\mathbf A\right);$$

$\:{\mathbf{e}}_{1}$ and $\:{\mathbf{e}}_{2}$ are the solution vectors of random residuals following normal distribution:

$$\:\begin{bmatrix}{\mathbf e}_1\\\:{\mathbf e}_2\end{bmatrix}\sim MVN\left(\begin{bmatrix}0\\\:0\end{bmatrix},\begin{bmatrix}{\sigma\:}_{{\text{e}}_1}^2&\:{\sigma\:}_{{\text{e}}_1,{\text{e}}_2}\\\:{\sigma\:}_{{\text{e}}_1,{\text{e}}_2}&\:{\sigma\:}_{{\text{e}}_2}^2\end{bmatrix}\otimes\:\mathbf I\right)\mathit.$$

The *summary* flag was used to view the variance components output by the model, all in ASReml-R.

### Genotypic data – quality control and GWAS-preprocessing

Fin clips were sent to IdentiGEN (Ireland) for DNA extraction through to genotyping using a ThermoFisher Axiom genotyping array containing 55,725 single nucleotide polymorphism (SNP) markers distributed evenly across the 27 chromosomes of the North American Atlantic salmon genome [32].

Standard quality control filtering was performed in PLINK v1.9 [33] (http://pngu.mgh.harvard.edu/purcell/plink/) according to the following criteria: retained loci with minor allele frequency (MAF) ≥ 0.05 (--*maf* flag), removed sites and individuals with > 10% missing data (--*geno* and --*mind* flags, respectively), removed individuals that had pi-hat values over 0.9 as they were likely duplicates (--*genome* flag), and filtered by Hardy-Weinberg Equilibrium using the --*hardy* flag. Also using pi-hat values, the relatedness of individuals was assessed. To further evaluate relatedness, a kinship matrix was generated using the *gVanRaden.2* function in the *gMatrix* package in R.

We computed principal components to visualize population stratification and also to use in the association analysis to correct for possible inflation of the association test statistics due to population stratification. In R, the *pcadapt* package [34] was used to compute principal components analysis (PCA), with an initial cluster (*K*) value of 50 and *min.maf* of 0.01. The *plot* function in R was used to visualize the data.

### Genome wide association

The package *ASRgwas* is a linear mixed model approach, which is used in tandem with *ASReml-R* to estimate genomic variance components and run the GWAS analysis. Genomic variance components estimates were obtained through the model generated using *gwas.asreml*, which is the same linear mixed model used for preliminary pedigree variance components estimation, except that a genomic relationship matrix (**G**) replaced the numerator relationship matrix (**A**).

Loci associated with FAs were identified using *ASRgwas*. Using the function *gwas.asreml*, the number of PC (*npc*) axes was determined from the screeplot (generated in the previous section) and the *p-*value threshold (*pvalue.thr*) for significant loci was set to 5.0e-6 and lowered until the false discovery rate was under 5% (evaluated at the end of each run). A suggestive significance threshold was also set to 2.0e-5. To determine the inflation factor (lambda or λ), the inverted distribution chi-squared function (*qchisq* in *snpStats* package) [35] was used to transform *p-*values into observed and expected values. Lambda was then calculated, which is the median of observed values divided by the median of expected values. Quantile-Quantile (QQ) plots were viewed using the *qqplot* function and Manhattan plots using the *manhattan* function, both in *qqman* in R [36].

### Gene region discovery

Once the gene regions of interest were determined from the GWAS, including a 100 K base pair (bp) region on either side of outlier SNP), Ensembl v.109 [37] and the North American *Salmo salar* assembly (USDA_NASsal_1.1, INSDC Assembly GCA 021399835.1) was utilized to find all associated genes. The Ensembl gene lists were then run in Metascape [38] (http://metascape.org) to generate a list of overlapping gene ontology (GO) terms.

## Results

### Family structure

**Fig. 1:**
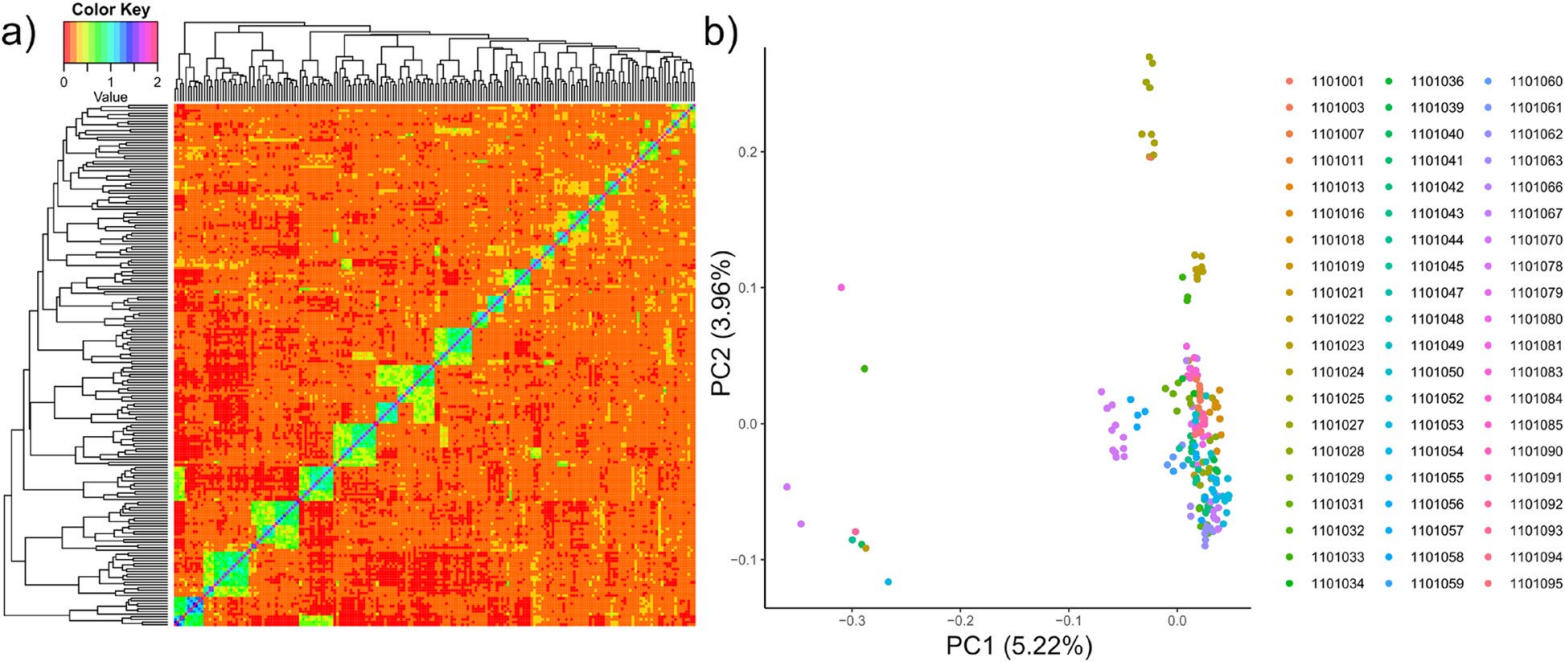
Family structure visualized by (**a**) kinship heatmap and (**b**) principal components analysis (PCA) which has been colored by families

A total of 208 individuals representing 60 families (1 to 14 individuals per family) were used in FA analysis. These are the individuals used hereon. There was an average of 0.0462 relatedness across all individuals and ranged from 0.000 to 0.701, based on pi-hat values from PLINK. A kinship heatmap revealed moderate levels of relatedness (sibling level) within families, but very low relatedness between different families (Fig. 1a), which was expected based on the partial factorial mating design. Based on the PCA plot, there were nine random individuals that were driving the majority of structure on axis one, in which the first axis was able to explain 5.55% of the variation in the data (Fig. 1b). Axis two revealed a relatively mixed group of families (3.96% of the variation, respectively; Fig. 1b). However, there were a few families that grouped together on the plot which may be families with slightly higher within-family relatedness than others.

### Fatty acids

Of the 35 FAs, 12 were n-3s, 8 were n-6s, 7 were SFAs, and 8 were MUFAs. The FAs that were present in the largest quantities based on mean percentages of total FA and the absolute amount in the tissue (mg/g of tissue), were palmitic acid (member of SFA; 16:0), oleic acid (member of MUFA; c9-18:1), and linoleic acid (member of PUFA; 18:2n-6) (Table 1; SI 1). These FAs combined made up for almost 70% of the FA content in the salmon fillet. Total MUFA were the largest FA group (ranged from 24.78 to 95.88 mg/g), followed by PUFA (ranged from 15.75 to 66.85 mg/g) and SFA (ranged from 8.408 to 30.88 mg/g) (Table 1). Total n-6 were higher than total n-3 FAs with a ratio of 1 n-3 to 1.31 n-6. There were 19 FAs with less than 0.5% of the total fatty acid content (SI 1). The distribution of residuals was evaluated and each FA was found to be normally distributed (SI 2 for an example of three FAs).

**Table 1:** Descriptive statistics of proportional and absolute content of selected fatty acids in muscle of North Atlantic salmon

Fatty acid		Proportional content (% of total FA)				Absolute content (mg FA/ 1 g muscle)			
		Mean	SE	Min	Max	Mean	SE	Min	Max
14:0	myristic acid	1.69	< 0.01	1.46	1.86	2.08	0.03	0.77	3.25
16:0	palmitic acid	11.2	0.03	10.3	12.1	13.76	0.20	5.65	20.84
c9-16:1	hypogeic acid	3.68	0.01	3.13	4.02	4.54	0.07	1.59	7.13
18:0	stearic acid	3.14	< 0.01	2.88	3.82	3.87	0.05	1.73	5.82
c9-18:1	oleic acid	41.3	0.04	39.9	42.9	50.73	0.72	20.91	80.36
c11-18:1/ 16:3n-3	vaccenic acid/hexadecatrienoic acid	2.79	< 0.01	2.62	2.91	3.43	0.05	1.37	5.51
18:2n-6	linoleic acid	16.9	0.03	15.9	17.7	20.77	0.30	8.08	33.13
18:3n-6	γ-linolenic acid (GLA)	0.35	< 0.01	0.18	0.63	0.43	< 0.01	0.15	0.75
11–20:1	gondoic acid	2.37	< 0.01	2.06	2.67	2.92	0.04	1.28	4.74
18:3n-3	alpha-linolenic acid (ALA)	3.50	0.01	3.03	3.85	4.30	0.07	1.54	7.13
18:4n-3	stearidonic acid (SDA)	0.62	< 0.01	0.44	0.85	0.765	0.01	0.25	1.24
20:2n-6	eicosadienoic acid	0.89	< 0.01	0.63	1.13	1.10	0.02	0.43	1.96
20:3n-6	dihimo-γ-linolenic (DGLA)	0.49	< 0.01	0.33	0.69	0.61	0.01	0.28	1.01
c11-22:1	erucic acid	1.13	< 0.01	1.01	1.29	1.39	0.02	0.62	2.20
20:4n-6	arachidonic acid	0.51	< 0.01	0.43	0.71	0.62	< 0.01	0.32	0.85
20:5n-3	eicosapentaenoic acid (EPA)	2.39	0.01	2.07	2.81	2.94	0.04	1.43	4.28
22:5n-3	docosapentaenoic acid (DPA)	0.97	< 0.01	0.77	1.14	1.20	0.02	0.54	1.81
22:6n-3	docosahexaenoic acid (DHA)	3.41	0.03	2.79	4.97	4.16	0.04	2.09	5.84
-	SFA	16.5	0.04	15.3	17.7	20.35	0.30	8.41	30.88
-	MUFA	49.2	0.04	48.0	50.7	60.54	0.86	24.78	95.88
-	PUFA	34.2	0.04	32.5	35.8	42.03	0.58	15.73	66.85
-	n-3	14.8	0.03	13.9	16.1	18.16	0.24	6.35	28.61
-	n-6	19.4	0.02	18.5	20.1	23.88	0.34	9.49	37.65

Only fatty acids with mean > 0.5 are presented with the exception of GLA and DGLA as they are important in the later results. SE is the standard error; *SFA* Saturated fatty acids, *MUFA* Monounsaturated fatty acids, *PUFA* Polyunsaturated fatty acids, *n-3* omega-3 fatty acids, *n-6* omega-6 fatty acids

### Fixed effects, phenotypic correlations and heritabilities

**Fig. 2:**
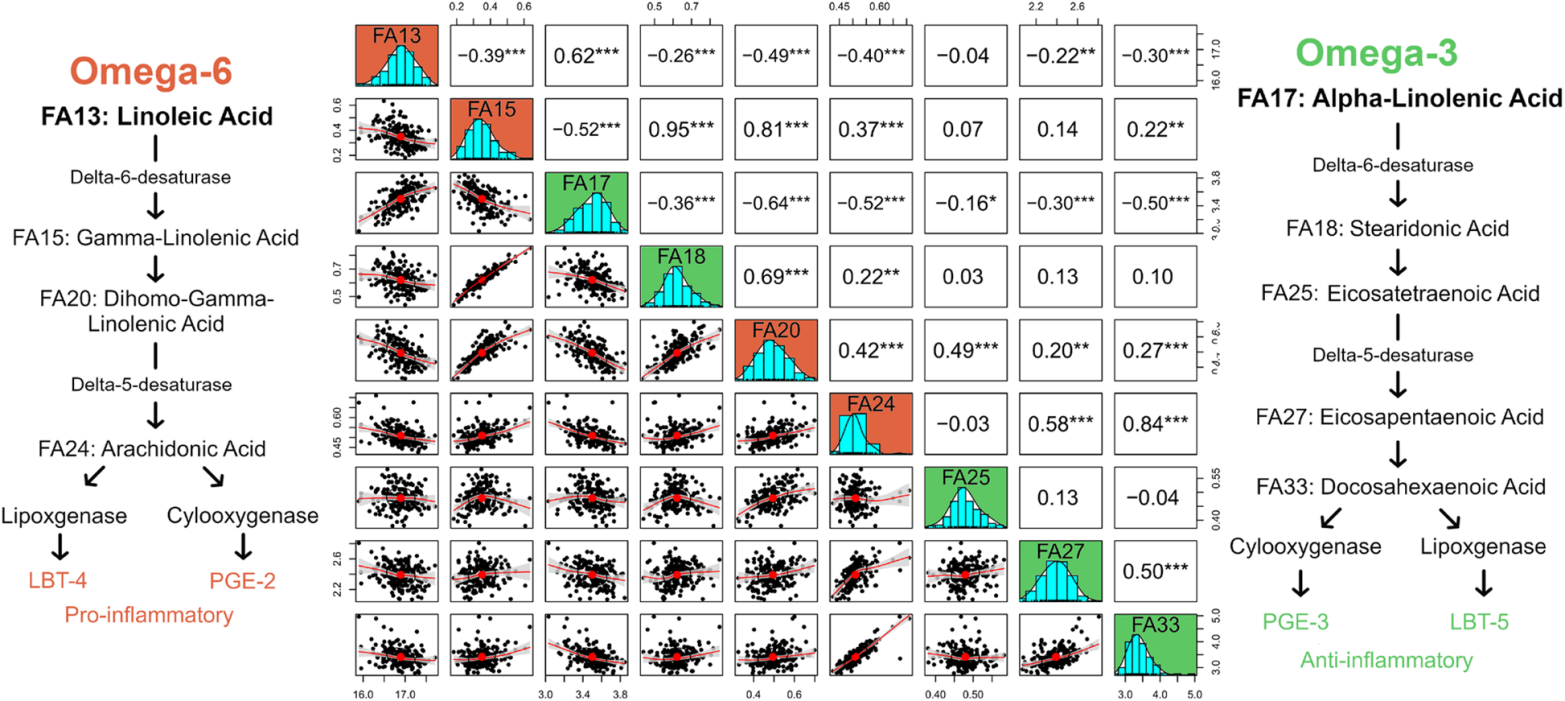
Pearson correlations between the major fatty acids involved in the omega-6 (n-6) and omega-3 (n-3) pathways. Values above the diagonal are Pearson correlations, bivariate plots are below the diagonal, and histograms are on the diagonal (where designations inside histograms correspond to fatty acids written in the pathways to the left or right of the plot). The color of the histogram corresponds to the omega pathway they belong to, where orange is omega-6 and green is omega-3. Significant correlations are designated by an ‘*’, where one indicates a *p*-value < 0.05, two indicates a *p*-value < 0.01, and three indicates a *p*-value < 0.001

Sex and maturity were both added as fixed effects and were significant for FAs, however they were not consistently significant across each trait (Table 2). When significant, fixed effects were added to models for genomic associations. Bled weight and total fat were highly correlated (genetic correlation) to FAs, at ~ 0.8 and ~ 0.9 respectively (Table 2). Many of the FAs had significant phenotypic correlations with other FAs (Fig. 2; SI 3; SI 4), however, there were a few with over 0.9: palmitic acid and SFA (correlation of 0.98), oleic acid and MUFA (correlation of 0.95), and γ-linolenic acid (18:3n-6) and stearidonic acid (18:4n-3) (correlation of 0.95). In general, FAs involved in the n-3 and n-6 pathways were moderately to highly correlated, with the highest correlations being between FAs that resided in the same position in their respective pathways (Fig. 2). For example, γ-linolenic acid from the n-6 pathway and stearidonic acid from the n-3 pathway had the highest correlations to each other, rather than to other FAs within the same pathway.

In general, genomic *h*^2^ estimates ranged from < 0.001 to 0.955 (Table 2), however most *h*^2^ estimates were moderate to high. The pedigree *h*^2^ estimates were mostly higher than the genomic *h*^2^ estimates (Table 2). The standard errors of the genomic *h*^2^ estimates were in general lower than that of the pedigree. However, due to the low sample size, the standard errors from both pedigree and genomic heritability estimate were generally high (> 0.10). For 15:0 and 22:5n-6 the *h*^2^ were estimated to be 0 (Table 2), while for 18:3n-6 and 20:3n-6 were estimated to be > 0.920 (Table 2;) for both genomic and pedigree information. The pedigree *h*^2^ for saturated (SFA) and unsaturated FAs (MUFA and PUFA) were estimated to be moderate. Pedigree *h*^2^ (SE) for SFA was 0.50 (0.17), while MUFA and PUFA had *h*^2^ (SE) of 0.29 (0.17) and 0.26 (0.14), respectively. The pedigree *h*^2^ (SE) of omega-3 and omega-6 fatty acids were 0.27(0.14) and 0.44(0.16), respectively.

**Table 2:** Descriptive statistics of the significance of fixed effects, genetic correlation with weight and total fat, pedigree and genomic heritability (*h*^2^) estimates, and their associated standard errors (SE) on the absolute content of fatty acid traits. Significant *p*-values (< 0.05) are indicated by ‘*’

Fatty acid	Sex *p*-value	Maturity *p*-value	Weight cor. ± SE	Total Fat cor. ± SE	Absolute content (mg FA/ 1 g muscle)	
					Pedigree h2 ± SE	Genotypic h2 ± SE
12:0	0.522	0.325	0.829 ± 0.115	0.956 ± 0.034	0.210 ± 0.148	0.120 ± 0.151
14:0	0.036*	0.055	0.882 ± 0.085	0.989 ± 0.006	0.321 ± 0.165	0.197 ± 0.147
15:0	0.058	0.884	0.884 ± 0.096	0.999 ± 0.000	< 0.001 ± 0.086	< 0.001 ± 0.001
16:0	0.519	0.263	0.878 ± 0.087	0.991 ± 0.005	0.469 ± 0.173	0.519 ± 0.152
c7-16:1	0.032*	0.447	0.845 ± 0.099	0.982 ± 0.009	0.635 ± 0.184	0.762 ± 0.146
c9-16:1	< 0.001*	0.002*	0.829 ± 0.102	0.992 ± 0.005	0.498 ± 0.177	0.297 ± 0.159
16:2n-3	0.015*	0.002*	0.843 ± 0.097	0.986 ± 0.008	0.429 ± 0.173	0.233 ± 0.152
17:0	0.543	0.209	0.909 ± 0.075	0.993 ± 0.008	0.066 ± 0.121	0.106 ± 0.156
c9-17:1	0.024*	0.002*	0.842 ± 0.101	0.988 ± 0.008	0.313 ± 0.154	0.160 ± 0.152
18:0	0.128	0.017*	0.865 ± 0.090	0.976 ± 0.011	0.773 ± 0.178	0.578 ± 0.144
c9-18:1	0.092	0.027*	0.800 ± 0.115	0.998 ± 0.000	0.282 ± 0.165	0.292 ± 0.179
c11-18:1/ 16:3n-3	0.143	0.635	0.828 ± 0.106	0.997 ± 0.000	0.260 ± 0.138	0.189 ± 0.163
18:2n-6	0.273	0.016*	0.805 ± 0.113	0.995 ± 0.000	0.615 ± 0.166	0.816 ± 0.133
20:0	0.745	0.116	0.811 ± 0.111	0.959 ± 0.023	0.536 ± 0.184	0.446 ± 0.177
11–20:1	0.208	0.339	0.811 ± 0.110	0.986 ± 0.007	0.602 ± 0.186	0.515 ± 0.165
18:3n-3	0.049*	0.059	0.757 ± 0.131	0.974 ± 0.012	0.804 ± 0.190	0.880 ± 0.122
18:3n-6	0.008*	0.513	0.350 ± 0.232	0.399 ± 0.198	0.924 ± 0.181	0.901 ± 0.131
18:4n-3	0.016*	0.869	0.640 ± 0.167	0.832 ± 0.075	0.793 ± 0.187	0.761 ± 0.157
20:2n-6	0.149	0.009*	0.812 ± 0.115	0.929 ± 0.033	0.752 ± 0.185	0.498 ± 0.148
20:3n-3	0.031*	0.002*	0.728 ± 0.146	0.870 ± 0.006	0.900 ± 0.183	0.528 ± 0.153
20:3n-6	0.002*	0.706	0.620 ± 0.171	0.661 ± 0.132	0.995 ± 0.182	0.955 ± 0.122
c11-22:1	0.853	0.413	0.803 ± 0.115	0.986 ± 0.008	0.318 ± 0.147	0.107 ± 0.152
c13-22:1	0.293	0.215	0.814 ± 0.123	0.999 ± 0.000	0.076 ± 0.127	0.281 ± 0.166
20:3n-3	0.031*	0.002*	0.728 ± 0.146	0.870 ± 0.006	0.900 ± 0.183	0.528 ± 0.153
20:4n-3	0.023*	0.690	0.850 ± 0.102	0.935 ± 0.033	0.597 ± 0.188	0.504 ± 0.171
20:4n-6	0.695	0.038	0.797 ± 0.117	0.921 ± 0.004	0.708 ± 0.190	0.583 ± 0.173
20:5n-3	0.327	0.918	0.861 ± 0.088	0.971 ± 0.016	0.441 ± 0.164	0.277 ± 0.303
21:5n-3	0.898	0.071	0.858 ± 0.091	0.998 ± 0.000	0.024 ± 0.096	0.131 ± 0.137
22:2n-6	0.034*	0.001*	0.790 ± 0.126	0.926 ± 0.041	0.484 ± 0.178	0.400 ± 0.153
22:4n-6	0.273	0.603	0.854 ± 0.093	0.979 ± 0.020	0.159 ± 0.135	0.300 ± 0.173
22:5n-3	0.001*	0.015*	0.887 ± 0.078	0.966 ± 0.019	0.461 ± 0.189	0.575 ± 0.174
22:5n-6	0.225	0.095	0.999 ± 0.000	0.999 ± 0.000	< 0.001 ± 0.001	< 0.001 ± 0.001
22:6n-3	0.465	0.091	0.804 ± 0.115	0.920 ± 0.040	0.497 ± 0.165	0.483 ± 0.163
24:5n-3	0.004*	0.034*	0.661 ± 0.166	0.664 ± 0.140	0.600 ± 0.170	0.516 ± 0.160
24:6n-3	0.198	0.994	0.504 ± 0.246	0.550 ± 0.212	0.331 ± 0.147	0.317 ± 0.153
c15-24:1	0.315	0.924	0.867 ± 0.114	0.951 ± 0.033	0.325 ± 0.165	0.370 ± 0.165
SFA	0.654	0.235	0.878 ± 0.086	0.991 ± 0.005	0.503 ± 0.174	0.504 ± 0.150
MUFA	0.315	0.387	0.806 ± 0.112	0.999 ± 0.001	0.291 ± 0.165	0.342 ± 0.178
PUFA	0.631	0.125	0.827 ± 0.105	0.997 ± 0.001	0.257 ± 0.143	0.331 ± 0.149
n-3	0.351	0.927	0.842 ± 0.099	0.996 ± 0.001	0.265 ± 0.139	0.187 ± 0.149
n-6	0.801	0.008*	0.814 ± 0.110	0.997 ± 0.001	0.436 ± 0.160	0.646 ± 0.151

*SFA* Saturated fatty acids, *MUFA* Monounsaturated fatty acids, *PUFA* Polyunsaturated fatty acids, *n-3* omega-3 fatty acids, *n-6* omega-6 fatty acids

### Fatty acids with significant loci from GWAS

**Fig. 3:**
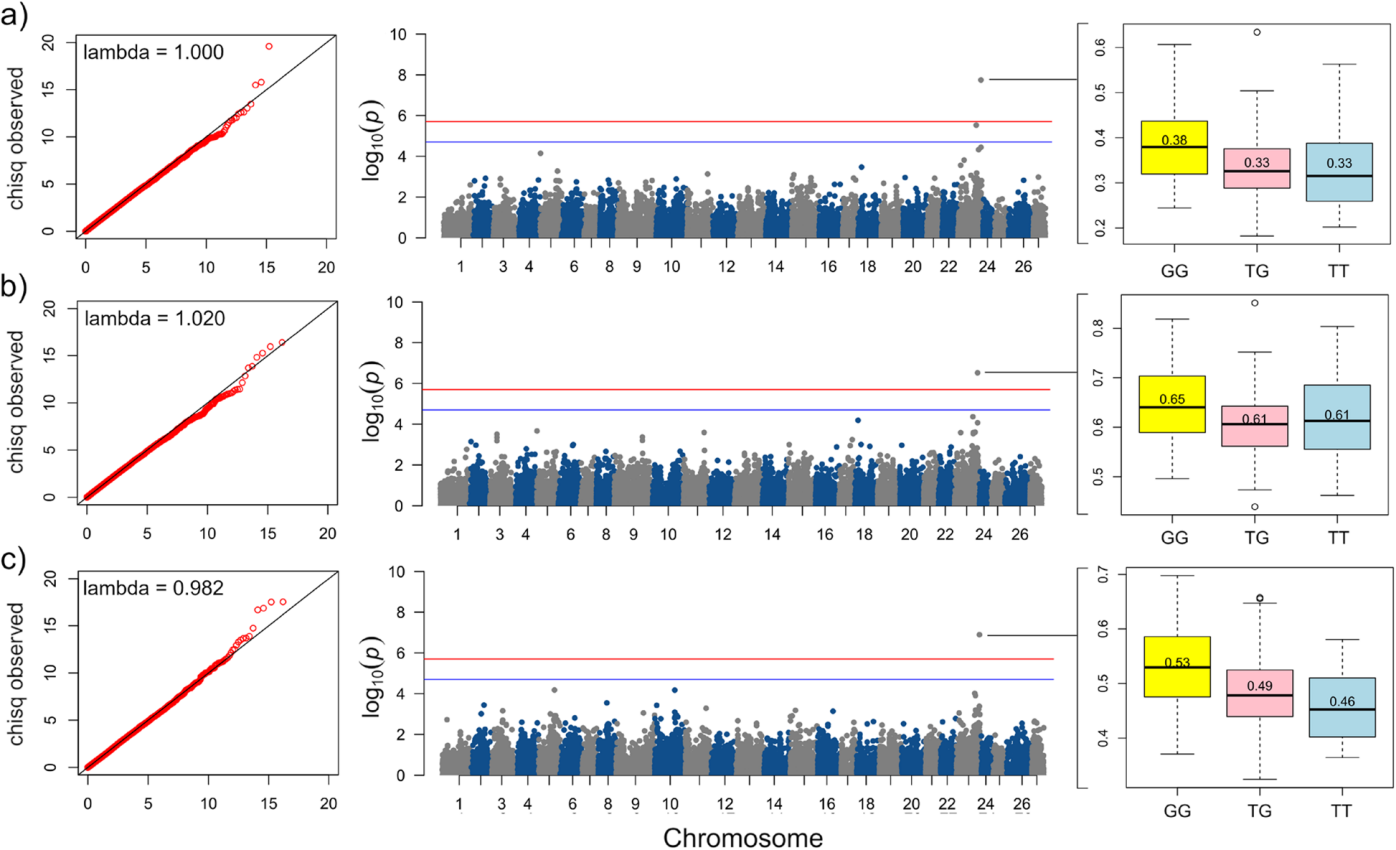
Manhattan plots of significant ASReml-genome wide associations from fatty acids: (**a**) γ-linolenic acid, (**b**) stearidonic acid, and (**c**) dihimo-γ-linolenic acid. The red line is a genomewide line set to 2.0e-6 and the blue line is a suggestive significant threshold set to 2.0e-5. QQplots with lambda values are in cut-outs on the left end of each Manhattan plot, where a lambda value close to 1.00 signifies little to no population stratification in the data. On the right are boxplots of the top significant SNP where the effect of alleles can be observed for each of the fatty acids. The three genotypes are GG, TG, and TT. The values listed on each box are the mean of each genotype

Of all the FAs analyzed, three PUFAs were found to have significant loci lower than the *p*-value threshold (2.0e-6). The only two significant markers for these three FAs (g-linolenic acid, stearidonic acid, and dihimo-g-linolenic acid (20:3n-6)) was located on chromosome 23 (75,495,237 and 94,789,779) (Fig. 3). The top locus for γ-linolenic acid, stearidonic acid, and dihimo-γ-linolenic acid was AX-520,351,564 (pos 94,789,779) and based on this singular locus, the G allele resulted in an increased FA content (Fig. 3). The A allele of the second top locus, AX-520,350,996 (pos 75,495,237), resulted in a higher value overall (SI 5). The top locus from these three FAs had an explained variance that ranged between 15 and 18%. There were an average of 5309 loci with over 1% of explained variance for these three FAs. Interestingly, the genetic correlations between all three FAs were moderate to high, ranging from 0.81 to 0.97 (SI 3). The genes found within 100 K bp of these two significant loci were: *xpr1a*, *haus5*, *sin3b*, *acbd6*, *lhx4*, *prtg*, *arpp19*, *myo5c*, *gnb5a*, *ap4e1*, *fads2*, and *cyp19a1*. The only gene ontology term connecting these genes (specifically including *sin3b*, *acbd6*, and *fads2*) was the metabolism of lipids.

### Fatty acids with loci only exceeding the suggestive threshold

Additionally, five other FAs had distinct peaks on Manhattan plots with loci crossing the suggestive threshold (*p*-value < 2e-05), however, without loci crossing the genomewide significance threshold: lauric acid (12:0), stearic acid (18:0), eicosatrienoic acid (ETE; 20:3n-3), eicosatetraenoic acid (ETA; 20:4n-3), and docosadienoic acid (22:2n-6; SI 6). Lauric acid had a peak at chromosome 2 (highest locus at position 53,835,328) which was within 100 K bp of three genes: *map4k3a*, *sos1*, and *tmem178*. Stearic acid had a peak at chromosome 4 (highest locus at position 16,416,473), which was within 100 K bp of *ints13*. ETE acid had the same loci as the FAs in the section above (chromosome 23, position 75,495,237). ETA had a peak on chromosome 10 (highest locus at position 105,082,055), which was within 100 K bp of two genes: *hipk2* and *mical3a*. Docosadienoic acid had a locus at chromosome 17 (not a peak) at position 11,462,317 and was associated with two genes: *mid1* and *arhgap6*. The top locus for each FA had an explained variance that ranged between 11 and 16%. There were an average of 5429 loci with over 1% of explained variance for these FAs.

## Discussion

The FAs are an essential part of a healthy functioning body system. However, in many cases FAs need to be acquired from diet. Therefore, knowing how much of each FA is stored in our food, for example fish, and how we can potentially make improvements through selection is of high economic value. In this study, GWAS was performed on 35 different FAs and five calculated traits (SFA, MUFA, PUFA, n-3 and n-6) in North American Atlantic salmon. Moderate to high *h*^2^ were found for many FAs, implying selection would be possible. While a distinct peak was observed in Manhattan plots at chromosome 23 for four FAs, the majority of other FAs did not have significantly associated loci. However, there were an additional five with distinct peaks at a variety of chromosomes, crossing the suggestive significance threshold. The only gene ontology term shared between the genes located at these peaks was the metabolism of lipids. Here, we provide a selection of candidate genes to further analyze in functional studies.

### Levels of fatty acids in the North Atlantic salmon fillet

The absolute amount of fatty acids in Atlantic salmon fillets were between 0.04 and 41.3 mg FA/g, which was a similar range as found in a previous study on Atlantic salmon (0.06 to 39.4 mg FA/g tissue) [39]. There were 19 fatty acids present in trace amounts, meaning they contributed less than 0.5% to overall fatty acid content. Palmitic acid (member of SFA), oleic acid (member of MUFA), and linoleic acid (member of PUFA) were present in the largest quantities (proportional and absolute amount) in the fish fillets: mean of 11.2, 41.3, and 16.9 mg FA/g tissue, respectively. A previous study by Colombo et al. [39] also found these three FAs to be present in the largest quantities in farmed Atlantic salmon that were sourced from a grocery store in Nova Scotia, Canada, with similar means of 12.6, 39.4, and 14.6 mg FA/g tissue, respectively. Within the same study, authors compared fatty acids of farmed Atlantic salmon to Chinook (wild and farmed), wild Pacific, and wild Sockeye salmon, and found farmed Atlantic salmon had the highest and/or most balanced amount of SFAs to MUFAs to PUFAs. The same three fatty acids listed above (palmitic acid, oleic acid, and linoleic acid) were also present in the highest quantities in beef (25.1, 37.9, and 1.87 respectively) [19] and sheep (1.54, 36.04, and 3.99 respectively) [40], with authors indicating these results were similar to other studies as well [41–44]. Based on these studies, Atlantic salmon appears to be a better source of PUFAs in general, than livestock animals and contains a more balanced intake of all fatty acids when compared to other fish species.

In terms of diet and health, salmon fillets are widely consumed for their n-3 long-chain-PUFAs, which are essential for human cardiovascular health, growth, immune system functions, and neurological health [45, 46], to name a few. However, it is more valuable in nutrition to consider the ratio of n-3 to n-6 given that a balance is essential for reducing the risk of developing many diseases such as cardiovascular diseases, various cancers, asthma, and osteoporosis, amongst others [47]. Within the literature there is a slight discrepancy in the ideal ratio of n-3: n-6, however it generally lands between 1:1 to 1:4 (summarized by Candela et al., [47]). In general, farmed Atlantic salmon will have a higher n-6 to n-3 ratio [than wild Atlantic salmon] as their diet contains more n-6 fatty acids, and a large portion of the fat in farmed salmon comes from n-6 fatty acids. Here, we report a ratio (using the means) of 1:1.3, which falls within the ideal ratio limits, similar to another recent study on Atlantic salmon which found a ratio of 1:1.5 [39]. As this is only a singular year class (2011 YC), it is possible that n-3 to n-6 ratios may be different in different year classes of fish, or there may be important year-by-year shifts (i.e., trends) in the ratio that we are unable to evaluate with this dataset but will be very important to the future of nutritional content of salmon. Changes to feed over time to increase sustainability and decrease feed costs while maintaining essential nutrition needed by the salmon may still result in excellent levels of essential omegas. Monitoring the relative balance of n-3 to n-6 as these changes occur will be important.

### Omega pathways and metabolism of lipids

Based on Pearson correlations between FAs from the n-3 and n-6 pathways (Fig. 2), as well as genomic peaks at the same position from FAs involved in both pathways, it is possible that the same genomic regions are associated with the causal genes required for these essential metabolic FAs. Previous research looking at the *h*^2^ of n-3 and n-6 FAs in cattle uncovered low to moderate results (*h*^2^ of 0.07 to 0.20) [41, 48], in pigs uncovered moderate to high *h*^2^ (0.21 to 0.89) [49, 50], and in poultry uncovered low to moderate results (0.116 to 0.310) [51], with research on n-3 FAs in European Atlantic salmon showing similar *h*^2^ to cattle (0.05 to 0.28) [4, 9, 52]. We have uncovered genomic *h*^2^ that were on average higher (n-3 *h*^2^ of 0.19 and n-6 *h*^2^ of 0.65) than most of these studies, which could be due to low sample sizes, stronger family structure, differences in diet, and age/life stage of the animal. It will be necessary to increase sample size and optimal family structure to obtain unbiased and precise *h*^2^ estimates. In addition, an increase of the number of SNPs in the array could determine if there are additional genomic regions that have been overlooked.

Previous GWAS research into all the different FA related genes in Atlantic salmon is limited. However several studies into the n-3 pathway found that FAD and ELOVL enzymes influence the content of n-3 LC PUFA in the liver [53–55], and can be found expressed in skeletal muscle tissue of Atlantic salmon [52, 56] Further research identified a promising gene for the ratio of DHA to DPA: *elovl2* on chromosome 21 [4]. Unfortunately, the authors could not find any significant SNPs associated directly with either DHA or EPA, however, they did identify suggestive peaks on chromosome 21. The genes identified in our study at the significant peak on chromosome 23 (where the three FAs were g-linolenic acid, stearidonic acid, and dihimo-g-linolenic acid) had one overlapping gene ontology term: metabolism of lipids. One gene that contributed to this GO term, and the most relevant here, was *fads2*, which has been previously identified as a significant gene in the n-3 pathway (see above) and is functionally also an important enzyme in the n-6 pathway. Fatty acid desaturase 2 or *fads*, is important in regulating the unsaturation of FAs by introducing double bonds between defined carbons of the FA chain (i.e. specifically, *fads2* catalyzes steps within the n-3 and n-6 pathways [57]). Therefore, it is not surprising that this gene was identified in the peak, and it is an excellent candidate for future functional studies.

The other two genes that contributed to the metabolism of lipids GO term, were *acbd6* and *sin3b*. The acyl-CoA-binding domain containing protein 6 (ACBD6) is important as a carrier and as a regulator of acyl-CoA dependent reactions, which help to control the lipid and protein composition in membranes of the cell [58]. Fatty acyl-CoA synthetases are essential in helping to convert long-chain FAs into very-long-chain FAs. In a recent GWAS study looking at quality traits in Duroc pigs, authors identified *cacna1e* and *acbd6* as the likely candidate genes involved in backfat thickness [59], while another study determined that milk fat from a variety of species was highly correlated to *acbd6* [60], implying that the *acbd6* gene may be fairly conserved across a variety of genera. Additionally, *acbd6* was found to be differentially expressed in chicken hepatocytes when folic acid was omitted or added to the culture medium [61]. Based on the involvement of *acbd6* in FA related traits, this gene may facilitate the identification of causal genes in these pathways in Atlantic salmon.

The *sin3b* gene encodes a paired amphipathic helix protein that acts as a transcription corepressor for many different genes [62]. In short, the mitochondrial organelle contains important proteins encoded by nuclear genes, of which many of these genes are regulated by the SIN3 transcriptional corepressor [63]. These proteins include those involved in enzyme synthesis such as those required for FA oxidation, oxidative phosphorylation, and removal of reactive oxygen species [64]. It is suggested that SIN3 has an evolutionarily conserved role in metabolism [65], therefore, also making the *sin3b* gene a good candidate for further exploration in Atlantic salmon.

### Fatty acid loci that cross the suggestive threshold

There were five FAs with clear peaks with loci that passed the suggestive significance threshold in Manhattan plots: lauric acid, stearic acid, ETE, ETA, and docosadienoic acid. The genomic *h*^2^ of these FAs were all moderate to high (0.12 to 0.58) but Pearson correlation generally low (ranged from − 0.04 to −0.23), despite many of them being part of the n-3 or n-6 pathway. This information, combined with the strong correlations between FAs found in the same position of omega pathways (listed above), imply the transformation process may be more similar (i.e. genes acting on each step of both pathways) and will be more genomically linked than overall pathways themselves. We further discuss the FAs with the most promising candidate genes below.

ETA is an intermediate FA produced within the n-3 pathway. We found moderate *h*^2^ in ETA (0.50), however a variety of *h*^2^ have been reported in rainbow trout (0.16 to 0.61) [66, 67]. In Atlantic salmon, a previous study that grouped PUFAs (ALA/EPA/DHA) found an *h*^2^ of 0.77 from a population size of ~ 1,200 individuals and 48 families [68], compared to cattle with an *h*^2^ of 0.08 [69] from ~ 1,000 individuals and eight different farms. Based on these results, it is possible that, like linoleic acid, ETA may vary genetically by breed and strain. ETA had a peak on chromosome 10 which was found associated with two genes, however, only one appeared relevant here: *hipk2*. The *homeodomain-interacting protein kinase 2* (*hipk2*) gene is a serine/threonine protein kinase that is involved with transcription regulation. This gene was found to be important in adipogenesis (i.e., the formation of fat cells from stem cells), specifically in white adipose cell differentiation and tissue development [70, 71].

Docosadienoic acid is an n-6 FA that is produced by the elongation of eicosadienoic acid, which is in turn produced by elongating GLA. Very little research has focused on docosadienoic acid, however one study on broiler chickens (i.e., chickens bred and raised for meat production) found this FA to be important in the differential expression between lean and fat birds and had a moderate heritability of 0.33 ± 0.12 [72]. Here, we found a similar *h*^2^ of 0.40 ± 0.15 and one significant locus on chromosome 17 associated with one relevant gene: *rho GTPase activating protein 6* (*arhgap6*). ARHGAP6 is important in the regulation of RhoA, leading to actin fiber depolymerization [73]. In Atlantic salmon, *arhgap6* was found to be positively correlated with *vgll3* which is an important age-at-maturity gene, implying *arhgap6* may play a role in maturation-related cellular process as well [74]. A GWAS on porcine meat samples identified ARHGAP6 within an important QTL for growth traits [75], which may be significant within the context of fat deposition.

### Remaining fatty acids

Out of all FAs, only three had a significant peak, while five had nonsignificant peaks (but that crossed the suggestive significance threshold). A previous GWAS study on individual FA in salmon, was unable to identify any specific regions or genes associated with FAs, therefore leading them to conclude that they were likely polygenic [4]. However, Horn et al. [4] only investigated a few genes from the n-3 pathway. While we found a similar result, we can expand to include most members of the n-6 pathway, as well as most SFAs and MUFAs. Previous studies in other species, such as beef and pork found that in general FA composition is a polygenic trait that is controlled by a few major and many minor genes [19, 76–80]. It is possible that Atlantic salmon is the same based on the results/candidate genes generated here, implying FA composition may be controlled by many genes of small effect but a few of larger effect (which would explain the distinct peaks that did not cross the genome-wide line). The lack of QTL identification for some of the FA could be due to the low sample size and therefore lack of power to help identify these QTLs. In the future it will be important to repeat this work using a larger sample size and increasing the investigation to include functional studies in order to confirm the exact connection of genes to the fatty acids.

## Conclusions

Most FAs had moderate to high *h*^2^, implying that selection for improvement of North American Atlantic salmon traits in the future could be possible. The ratio of n-3 to n-6 was within the ideal range (1:1.3). This study provides an important baseline for future tracking to ensure the ideal range is maintained and total omega values are maintained while diets are improved to increase sustainability and decrease production costs. Three FAs had the same genomic peak on chromosome 23, which contained three genes (*sin3b*, *acbd6*, and *fads2*) that were all involved with the metabolism of lipids, making these genes ideal candidates for future functional studies.

## Supplementary Information

Supplementary Material 1.

Supplementary Material 2.

## Data Availability

The genetic data and metadata are included as supplementary information.
