# Supplementary material for: Candidate genes associated with fatty acid compositions in north American Atlantic salmon (Salmo salar)
Source: BMC Genomics. 2024 Dec 18;25:1208. doi: 10.1186/s12864-024-11131-2 (PMC11658282; doi:10.1186/s12864-024-11131-2)
Supplement: Supplementary file 2 — Supplementary Material 2. [file 12864_2024_11131_MOESM2_ESM.docx]

SUPPLEMENTARY DATA

SI 1: Descriptive statistics of proportional and absolute content of selected fatty acids in muscle.

| Fatty acid | | Proportional content (% of total FA) | | | | Absolute content (mg FA/ 1 g muscle) | | | |
| --- | --- | --- | --- | --- | --- | --- | --- | --- | --- |
|  |  | Mean | SE | Min | Max | Mean | SE | Min | Max |
| 12:0 | Lauric | 0.03 | <0.01 | 0.02 | 0.04 | 0.04 | <0.01 | 0.01 | 0.06 |
| 15:0 | Pentadecanoic | 0.15 | <0.01 | 0.12 | 0.17 | 0.19 | <0.01 | 0.07 | 0.28 |
| c7-16:1 | Palmitoleic | 0.28 | <0.01 | 0.25 | 0.31 | 0.35 | <0.01 | 0.14 | 0.53 |
| 17:0 | Heptadecanoic | 0.13 | <0.01 | 0.12 | 0.16 | 0.16 | <0.01 | 0.07 | 0.26 |
| c9-17:1 | Oleopalmitic | 0.10 | <0.01 | 0.08 | 0.13 | 0.13 | <0.01 | 0.05 | 0.22 |
| 16:2n-3 | Hexadecadienoic | 0.22 | <0.01 | 0.19 | 0.25 | 0.28 | <0.01 | 0.10 | 0.44 |
| 20:0 | Arachidic | 0.21 | <0.01 | 0.17 | 0.25 | 0.26 | <0.01 | 0.12 | 0.39 |
| 18:3n-6 | γ-linolenic (GLA) | 0.35 | <0.01 | 0.18 | 0.63 | 0.43 | <0.01 | 0.15 | 0.75 |
| 20:3n-6 | dihimo-γ-linolenic (DGLA) | 0.49 | <0.01 | 0.33 | 0.69 | 0.61 | 0.01 | 0.28 | 1.01 |
| c13-22:1 | Gondoic | 0.24 | <0.01 | 0.19 | 0.33 | 0.29 | <0.01 | 0.12 | 0.46 |
| 20:3n-3 | Eicosatrienoic (ETE) | 0.19 | <0.01 | 0.13 | 0.26 | 0.23 | <0.01 | 0.08 | 0.43 |
| 20:4n-3 | eicosatetraenoic (ETA) | 0.48 | <0.01 | 0.38 | 0.58 | 0.59 | <0.01 | 0.24 | 0.89 |
| 22:2n-6 | docosadienoic | 0.09 | <0.01 | 0.06 | 0.12 | 0.11 | <0.01 | 0.04 | 0.19 |
| c15-24:1 | Nervonic | 0.16 | <0.01 | 0.13 | 0.21 | 0.19 | <0.01 | 0.08 | 0.29 |
| 22:4n-6 | Adrenic | 0.09 | <0.01 | 0.07 | 0.12 | 0.12 | <0.01 | 0.05 | 0.18 |
| 21:5n-3 | heneicosapentaenoic (HPA) | 0.13 | <0.01 | 0.09 | 0.15 | 0.16 | <0.01 | 0.06 | 0.25 |
| 22:5n-6 | Osbond | 0.09 | <0.01 | 0.06 | 0.14 | 0.12 | <0.01 | 0.04 | 0.17 |
| 24:5n-3 | tetracosapentaenoic | 0.06 | <0.01 | 0.02 | 0.11 | 0.07 | <0.01 | 0.02 | 0.18 |
| 24:6n-3 | tetracosahexaenoic | 0.03 | <0.01 | 0.02 | 0.06 | 0.04 | <0.01 | 0.02 | 0.11 |

Only fatty acids with mean < 0.5% are presented.


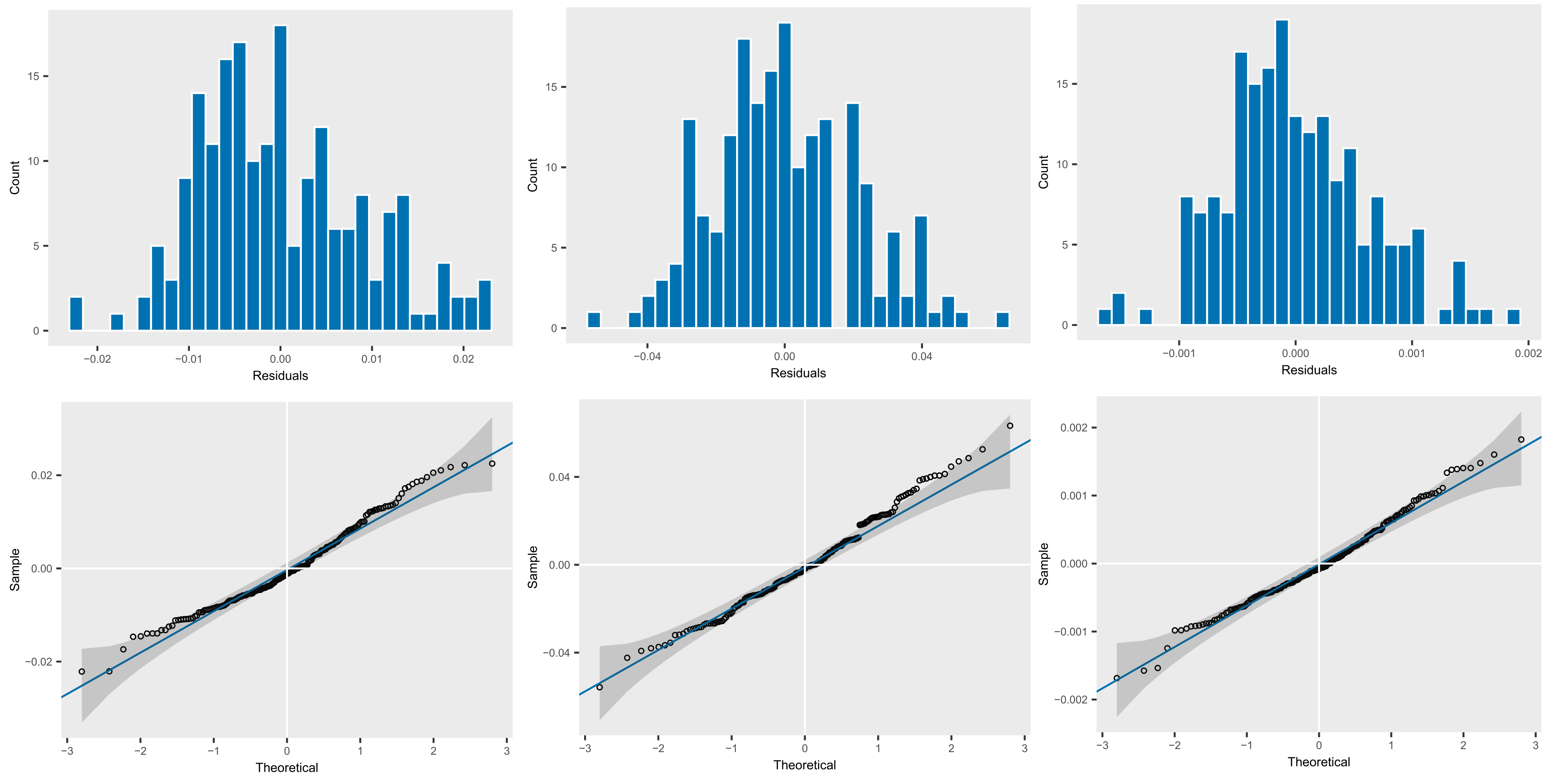


SI 2: Example plots showing the residuals of samples for fatty acids: γ-linolenic acid, stearidonic acid, and dihimo-γ-linolenic acid.

SI 3: Phenotypic and genetic correlations of all fatty acids. (excel file)


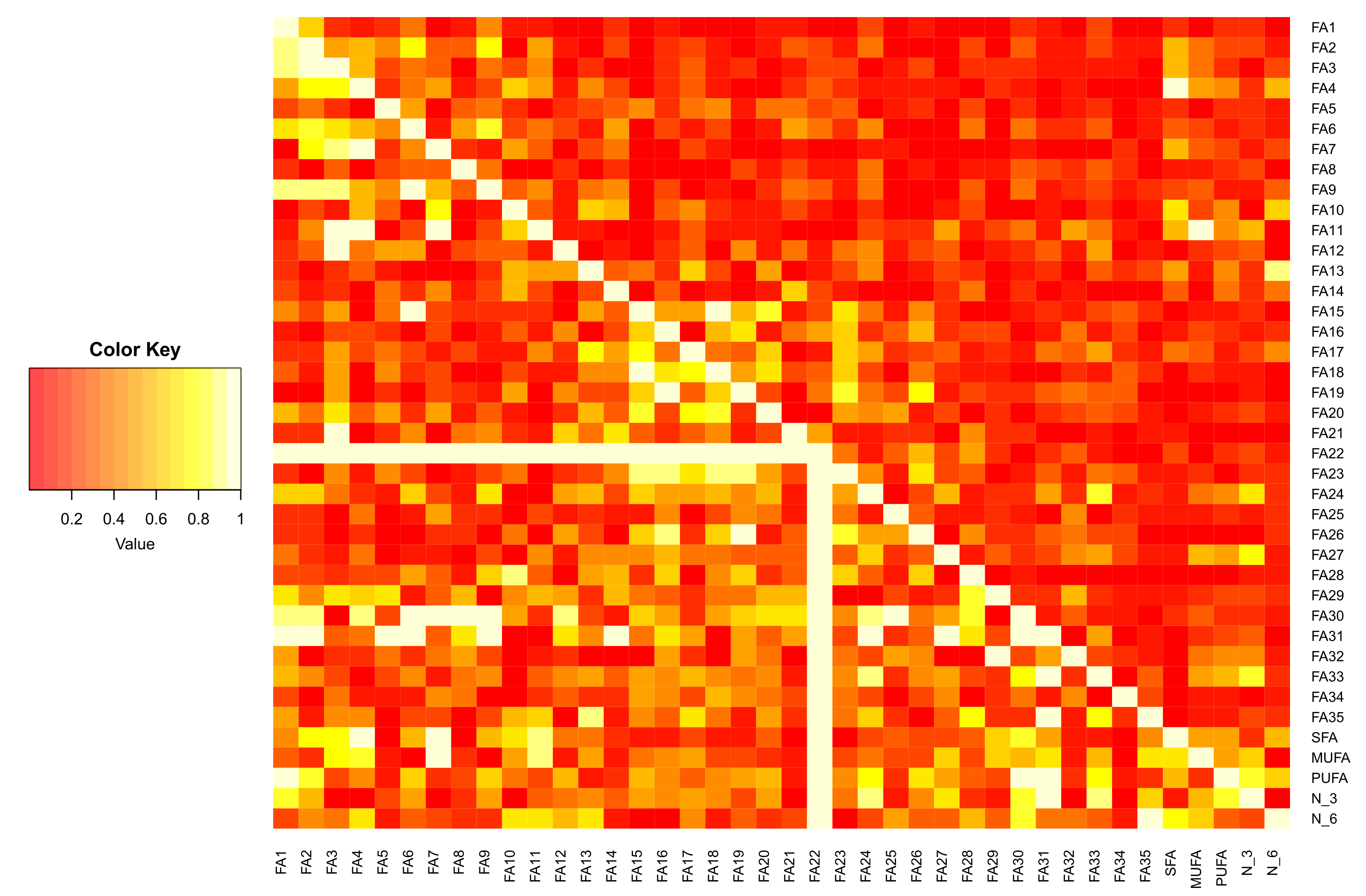


SI 4: Heatmap of all correlations between fatty acids where values above the diagonal are Pearson correlations between phenotypes, and values below the diagonal are genetic correlations.


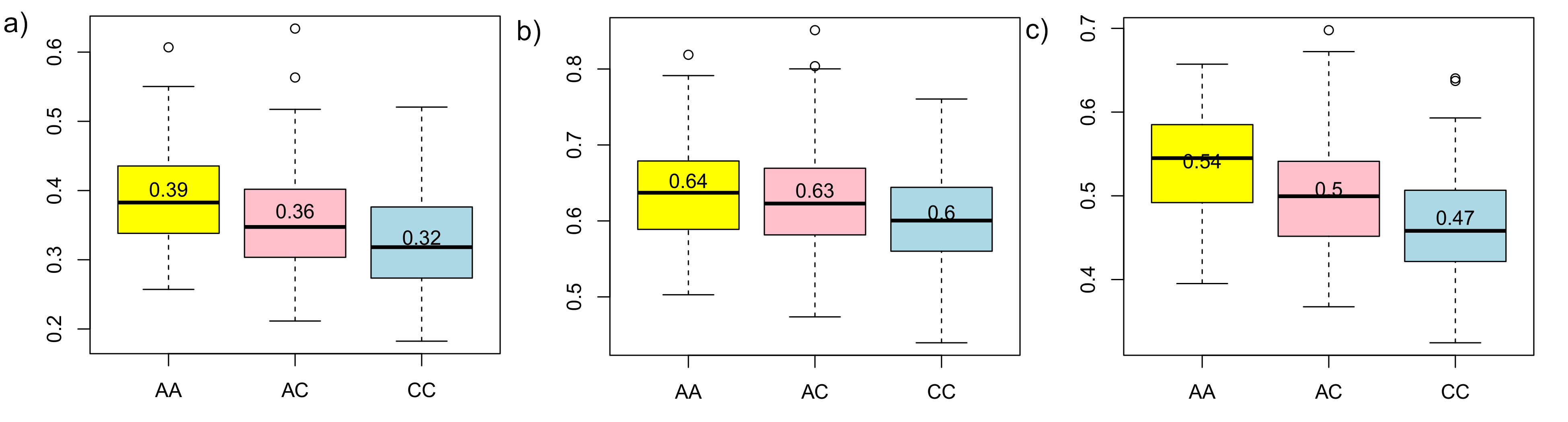


SI 5: Boxplots of the second top significant locus for a) γ-linoleic acid, b) stearidonic acid, and c) dihimo-γ-linolenic acid traits.


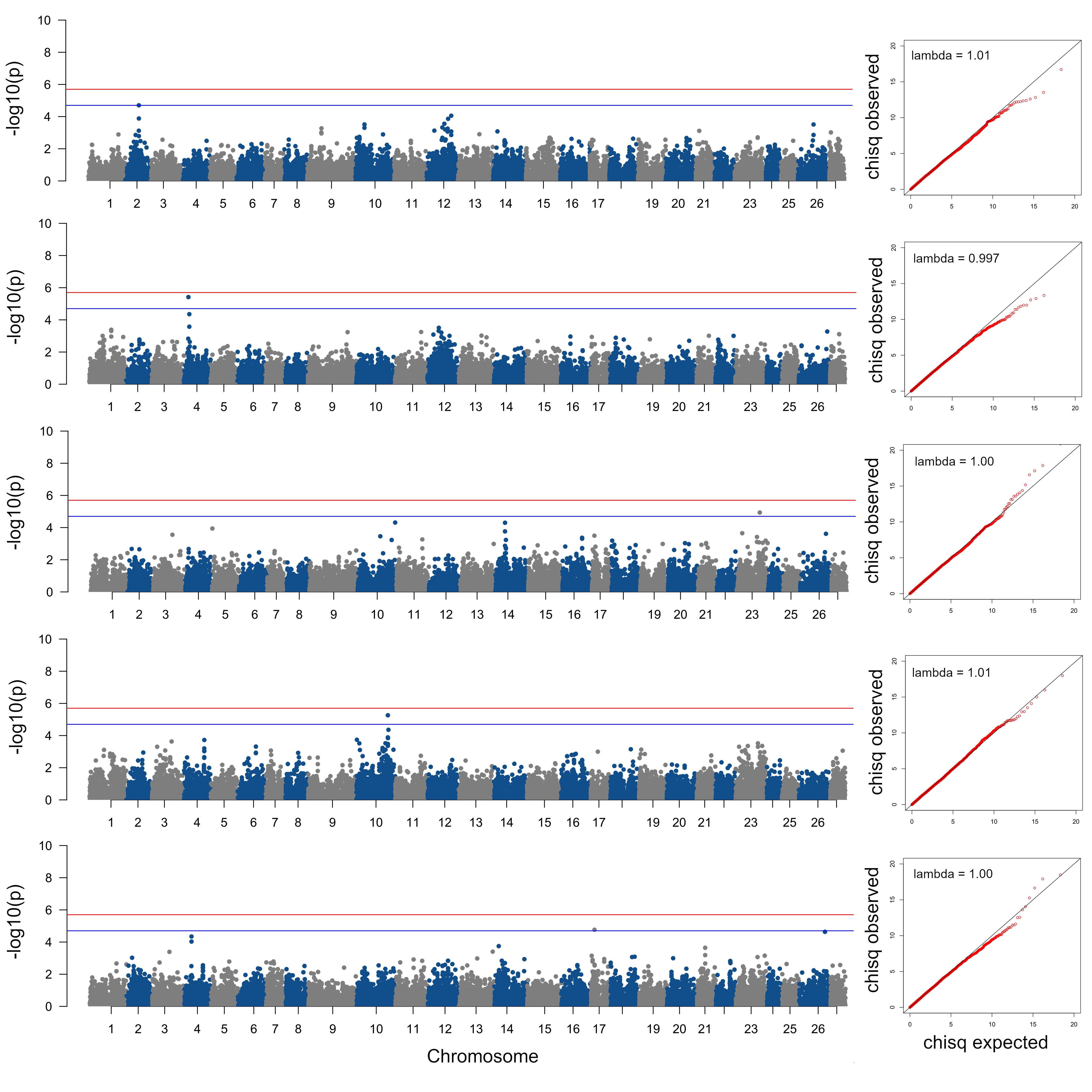


SI 6: Manhattan plots of ASReml-genome wide associations from fatty acids: lauric acid, stearic acid, eicosatrienoic acid, eicosatetraenoic acid (ETA), and docosadienoic acid. The red line is a genomewide line set to 2.0e-6 and the blue line is the suggestive significance threshold set to 2.0e-5. QQplots with lambda values are in cut-outs on the right end of each Manhattan plot, where a lambda value close to 1.00 signifies little to no population stratification in the data.
